# A scale to measure perceived respiratory effort in dogs: the DeChant scale

**DOI:** 10.3389/fvets.2025.1528357

**Published:** 2025-05-15

**Authors:** Mallory T. DeChant, Alexandra Moesta, Nathaniel J. Hall

**Affiliations:** ^1^Department of Animal and Food Sciences, Texas Tech University, Lubbock, TX, United States; ^2^Royal Canin Research and Development Center, Aimargues, France

**Keywords:** perceived respiratory effort, Labrador retriever, exercise paradigm, exercise induced hyperthermia, categorical psychophysical scale

## Abstract

The perceived respiratory effort (PRE) scale is a categorical psychophysical scale originally developed by Gunnar Borg and modified for numerous applications. We here propose a modification of the PRE scale with a 0–10 categorical scale for dogs, called the DeChant scale. A total of seventy-nine Labrador Retrievers were scored by video using the developed scale pre and post one of two different sprint exercise paradigms. The first exercise paradigm was 200 m in length and the second exercise paradigm was 1,200 m in length. PRE was reliably scored with an interclass correlation exceeding 0.8 for both exercise paradigms. The scale was further validated with moderate (*r* > 0.5) to strong correlations (*r* > 0.7) with core body temperature, rectal temperature, heart rate and respiration rate. The results suggest this PRE scale may be a useful, rapid and reliable visual measure of canine effort under exercise. Future research is needed for validation to other dog breeds and for use as a measure to predict detection performance or heat injury risk.

## 1 Introduction

The average core body temperature of dogs ranges from 37.5 to 38.8°C (1), however, during physical activity working dogs have been reported to reach higher rectal temperatures. For example, after strenuous exercise in Labrador Retrievers, dogs' rectal temperature reached 41.8°C (2) and search and rescue dog's internal temperature averaged 39.4°C during search work (3). Similarly, a peak core body temperature of 42.9°C was reported after exercise in working dogs (4). Exercise-induced hyperthermia would limit the capabilities of working dogs and may progress to heat injury or heat stroke. To mitigate the risk of heat injury or heat stroke, handlers routinely monitor the dog for signs of physical exertion or hyperthermia, however, currently there are no quantitative methods beyond temperature measurements.

The perception of effort refers to the sensation of how challenging and strenuous a physical task is (5). In regard to respiratory effort, the perception depends mainly on the feelings of effort and sensation of heavy breathing (5). The perceived respiratory effort (PRE) scale developed by Gunnar Borg (6, 7) is a psychophysical scale, i.e., it describes the relationship between a participant's perception and the physical intensity the person is subjected to (8). It is also a category-ratio (CR) scale, a scale where categories have the same distances between the scale values (8). The Borg PRE scale has been used in humans to rate perceptual intensity, physical performance and work capacity with 10 categories (Table 1) (8–11).

**Table 1:** CR-10 Borg Scale with description from Borg (9).

**Score**	**Level of exertion**
0	No exertion at all
0.5	Very, very slight (just noticeable)
1	Very slight
2	Slight
3	Moderate
4	Somewhat severe
5	Severe
6	
7	Very severe
8	
9	Very, very severe (almost maximal)
10	Maximal

The CR-10 Borg scale has been extensively validated in humans. Scores increase linearly with physical speed during exercise and with exercise-induced heart rate changes in humans (8, 10, 12, 13). It further allows for quantification of the effects of certain medical conditions on respiratory effort under light exercise, such as heart failure (14), chronic obstructive pulmonary disease (15), general dyspnea (16) or Parkinson's disease (13). The CR-10 Borg scale has also been used in the absence of exercise to assess the impact of respiratory conditions on perceived respiratory effort (17) and to monitor respiratory effort or workload in patients with neurological [e.g., spinal cord injury (18)], muscular [e.g., dystrophia myotonica (19)], or cardiovascular disease [e.g., recent hemispheric stroke (28)]. Over decades, the CR-10 Borg scale has been adjusted to fit the needs of different studies to successfully quantify perceived respiratory effort in humans [e.g., (29)].

There is only one study in dogs that has developed or utilized a psychophysical scale (20). They developed a perceived exertion scale for pet dogs of multiple breeds exercising on a treadmill, ranging from 0 to 4 where 0 was “no effort” and 4 was “significant effort”. This scale showed good inter-observer agreement and moderate correlation with physiological parameters such as glucose and cutaneous oximetry. The authors used light intensity exercise on a treadmill (i.e., the dogs walked or trotted at 2–4 mph at 0° incline). For working dogs, however, many of the activities required are of much higher intensity, creating a need for a scale with a greater range to be able to evaluate moderate to significant effort. The aim of this study therefore was to develop a 0–10 category ratio respiratory effort scale for dogs, the DeChant scale, based on an adaption of the CR-10 Borg Scale and evaluate its correlation with physiological parameters under different exercise conditions.

## 2 Materials and methods

### 2.1 Animals

A total of seventy-nine healthy Labrador Retrievers (average age 1.7 ± 0.75 SD years, 46 intact females, 1 spayed female, 30 intact males, 3 neutered males) participated in two separate exercise paradigms (Exercise Paradigm 1 and Exercise Paradigm 2), during which the DeChant perceived respiratory effort scale was evaluated. All dogs were privately owned and housed in a working dog facility in the Southern United States. Dogs that were included in both exercise paradigms were considered physically fit, not pregnant, received regular exercise, and were deemed healthy by the owner. The exercise paradigms were approved by Texas Tech University Institutional Animal Care and Use Committee IACUC #21001-01 and the Royal Canin Ethical Review Board (#220920-38, 091121-44).

### 2.2 Exercise paradigm 1

Forty-four dogs participated in a short sprint exercise. Each dog sprinted a 100 m lure course two times for a total of 200 m. After the first sprint, the dog was walked back to the starting point and immediately released for the second sprint. Dogs completed three sprint exercises in total (qualification, sprint test 1, sprint test 2). Forty-four dogs completed the first exercise session (qualification) in August 2021. Four dogs did not pass the qualifying criteria (i.e., they did not chase the rag on the lure) and were excluded from the sprint test 1 and 2. The remaining forty dogs completed the other two exercise sessions (sprint test 1 and sprint test 2) three months later in December 2021. Sprint test 1 and sprint test 2 were conducted on consecutive days in December 2021.

### 2.3 Exercise paradigm 2

Forty-four dogs participated in a long-distance sprint that consisted of the dog running back and forth 12 times running after a toy (i.e., Wubba toy™, Kong^®^, or ball depending on dog's toy preference) over a 100 m distance, totaling 1,200 m. Nine dogs that participated in Exercise Paradigm 2 had also participated in Exercise Paradigm 1. Dogs completed three long-distance sprint exercises in total (qualification, recall test 1, recall test 2).

A qualification run was conducted with 44 dogs over one day in August 2022, to ensure dogs were interested in running after a toy. Four dogs did not qualify (i.e., they did not want to run after one of the toys) and were excluded from the recall test 1 and 2. Forty dogs completed the remaining two exercise sessions (recall test 1 and recall test 2, over two consecutive days) in December 2022.

### 2.4 Diets

Diet A was fed to dogs participating in Exercise Paradigm 1 and Diet B was fed to dogs participating in Exercise Paradigm 2.^1^

### 2.5 Physiological measures

**Figure 1:**
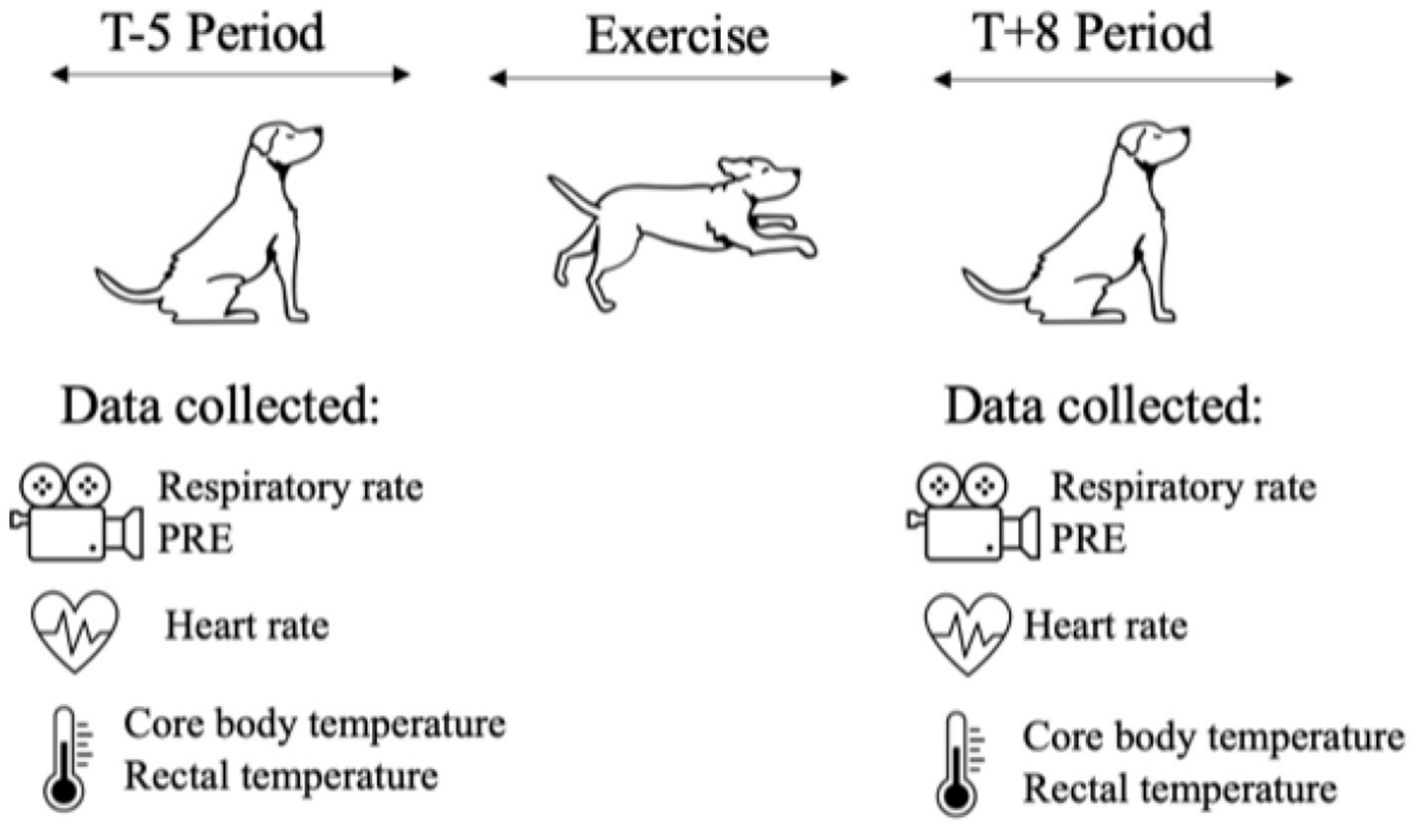
Graphic of the physiological measures collected during the two different time periods of each exercise paradigm. The video recording was utilized to determine respiratory rate and score PRE level once during the T-5 Period and once during the T+8 Period. Heart rate was recorded manually via femoral artery once during T-5 Period and once during T+8 Period. Core body temperature was recorded continuously every 10-s and then averaged for one measurement during the T-5 Period and one measurement during the T+8 Period. Rectal temperature was recorded once during T-5 Period and once during the T+8 Period.

Physiological measures were collected during two different time periods for each exercise paradigm: 5-min prior to the exercise paradigm until start of the exercise (T-5) and 8-min after the end of the exercise paradigm for 5 min (T+8) (Figure 1).

Data was collected in the following order: PRE score, respiratory rate, heart rate, and rectal temperature.

A 5-min video was recorded for each dog during the T-5 period and during the T+8 period for PRE score (Figure 1). The video was a lateral view of the dog's face and body. One author of this paper (DeChant) coded 100% of videos for exercise paradigm 1 and exercise paradigm 2. A second observer double-coded 100% of videos for exercise paradigm 1, and a third observer double-coded 20% of videos for exercise paradigm 2. A fourth observer triple-coded 20% of videos for exercise paradigm 1 and triple-coded 20% of videos for exercise paradigm 2 for inter-observer agreement. The second, third, and fourth observers were trained by the first author utilizing sample videos that are published in the supplementary section then clarified any questions the observers had on scores and finally were given the data set to be score.

Heart rate was taken via the femoral artery once during the T-5 period and once during the T+8 period. Rectal temperature was recorded once during the T-5 period and once during the T+8 period (Figure 1). Environmental temperature and humidity were recorded for each dog at the start of T-5 (Figure 1).

Core body temperature was recorded every 10 s using CorTemp Sensor capsules (HQInc^®^, Palmetto, FL, USA) (Figure 1). CorTemp Sensor capsules were given orally to each dog at least 30 min prior to exercise. The 10-s-epochs were averaged to calculate one core body temperature for the T-5 and T+8 periods.

### 2.6 Development of the scale

Adaptations to the CR-10 Borg scale were made based on physiological behaviors common in dogs while resting and exercising (Table 2). Examples of PRE scores 2–8 videos are attached in the Supplementary material.

**Table 2:** The DeChant scale of perceived respiratory effort.

**Unit measurement**	**Perceived respiratory effort**	**Description**
0	Nothing at all	Dog is resting and calm. Respiration is an involuntary process.
1	Very light	Dog is resting and calm. Respiration is greater than involuntary process; however, the chest does expand and collapse slightly more noticeably.
2	Light	Dog is not panting and is calm. The chest expands and collapses more noticeably to get more oxygen into lungs.
3	Moderate	Dog is not panting and is slightly aroused. The chest expands and collapses with greater momentum; however, abdomen is not engaged in respiration.
4	Somewhat heavy	Dog is panting where the tongue is visible and still retracted inside mouth. The dog is aroused. The chest expands and collapses with moderate effort and abdomen is slightly engaged in respiration.
5	Heavy	Dog is panting where the tongue is extended, and base of tongue is equal in surface area as body of tongue. The dog is aroused. The chest expands and collapses with moderate effort and abdomen is actively engaged in respiration.
6	Moderately heavy	Dog is panting where the tongue is extended, and base of tongue is more bulbous. The dog is aroused. The chest expands and collapses with great effort and abdomen is actively engage in respiration.
7	Very heavy	Dog is panting heavily where the tongue is fully extended, base of tongue is bulbous, and evaporative liquid is gathering and dripping. Moderately salivating. The chest expands and collapses with maximal effort and abdomen is contracting greatly.
8	Very very heavy	Dog is panting heavily where tongue is fully extended and lolling out to the side, base of tongue is bulbous, and evaporative liquid is dripping with excess foam collecting. Moderately salivating. The chest expands and collapses with maximal effort and abdomen is contracting greatly.
9	Submaximal	Dog is panting heavily where the tongue is fully extended and lolling out to the side, base of tongue is bulbous, and evaporative liquid is dripping with excess foam collecting. Heavily salivating. The chest expands and collapses with violent effort and abdomen is contracting violently. Audible wheezing/whistling sound when dog exhales.
10	Maximal	Dog is panting heavily with force where tongue is fully extended and lolling out to one side, base of tongue is bulbous, and evaporative liquid is dripping heavily. Heavily salivating. Tongue is discolored (dark red) from normal coloration. The chest expands and collapses with violent effort and abdomen is contracting violently. Audible wheezing/whistling sound when dog exhales. Dog is restless or agitated.

### 2.7 Statistical analysis

Analysis was separated for each Exercise Paradigm. A correlation matrix was computed between each physiological measure and PRE using the Performance Analytics package in R (27). To assess how a change in PRE score was associated with changes in physiological and environmental measures (core body temperature, rectal temperature, heart rate, respiratory rate, environmental temperature, and environmental humidity), generalized linear mixed-effect models were fit where PRE score predicted the physiological outcome measure of interest. Core body temperature data that was 36.11°C or lower was filtered out of the dataset due to the dog drinking cold water or interference with the sensor. Statistical significance of fixed effects was evaluated using the Anova function in the car package in R (21). The Interclass Correlation (ICC) package (22) in R was utilized for interobserver agreement. The lmer package (23) in R [R version 3.5.1, www.r.project.org; (30)] was used to fit models.

## 3 Results

### 3.1 Exercise paradigm 1

**Figure 2:**
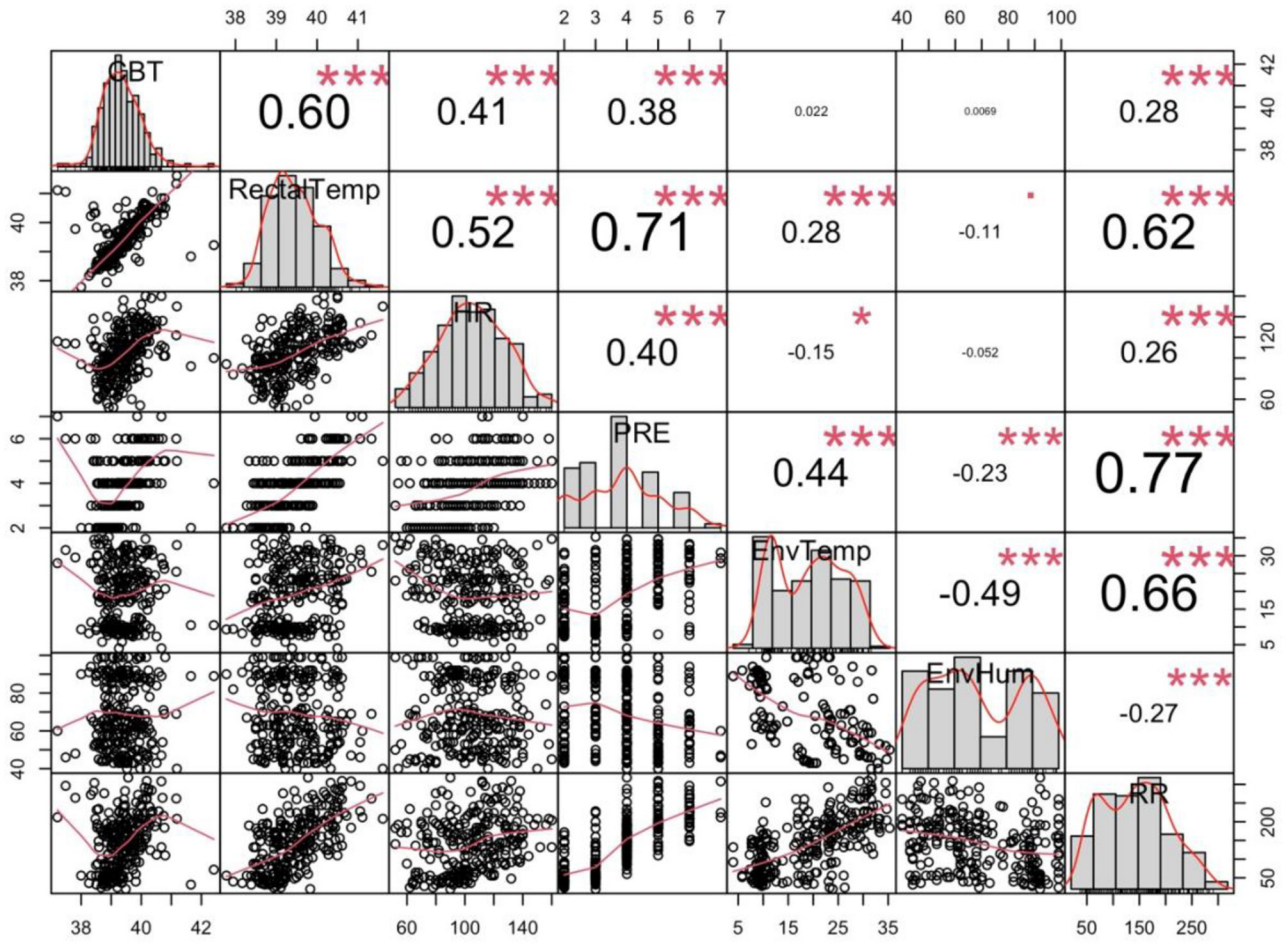
Correlation matrix of all data collected during Exercise Paradigm 1. Average core body temperature (CBT—°C), rectal temperature (RectalTemp—°C), heart rate, (HR—bpm), environmental temperature (EnvTemp—°C), environmental humidity (EnvHum—%), perceived respiratory effort score (PRE), respiratory rate (RR—bpm Significance level: *p ≤ 0.05, ***p < 0.001.

The interclass correlation for 100% of the double coded videos was 0.87 and for 20% of the triple coded videos was 0.84, indicating good reliability. A correlation matrix for data collected during Exercise Paradigm 1 is presented in Figure 2. There was a weak correlation between PRE score and core body temperature (*r* = 0.38), heart rate (*r* = 0.40), and environmental temperature (*r* = 0.44). There was a moderate correlation between PRE and rectal temperature (*r* = 0.71) and respiratory rate (*r* = 0.77).

**Figure 3:**
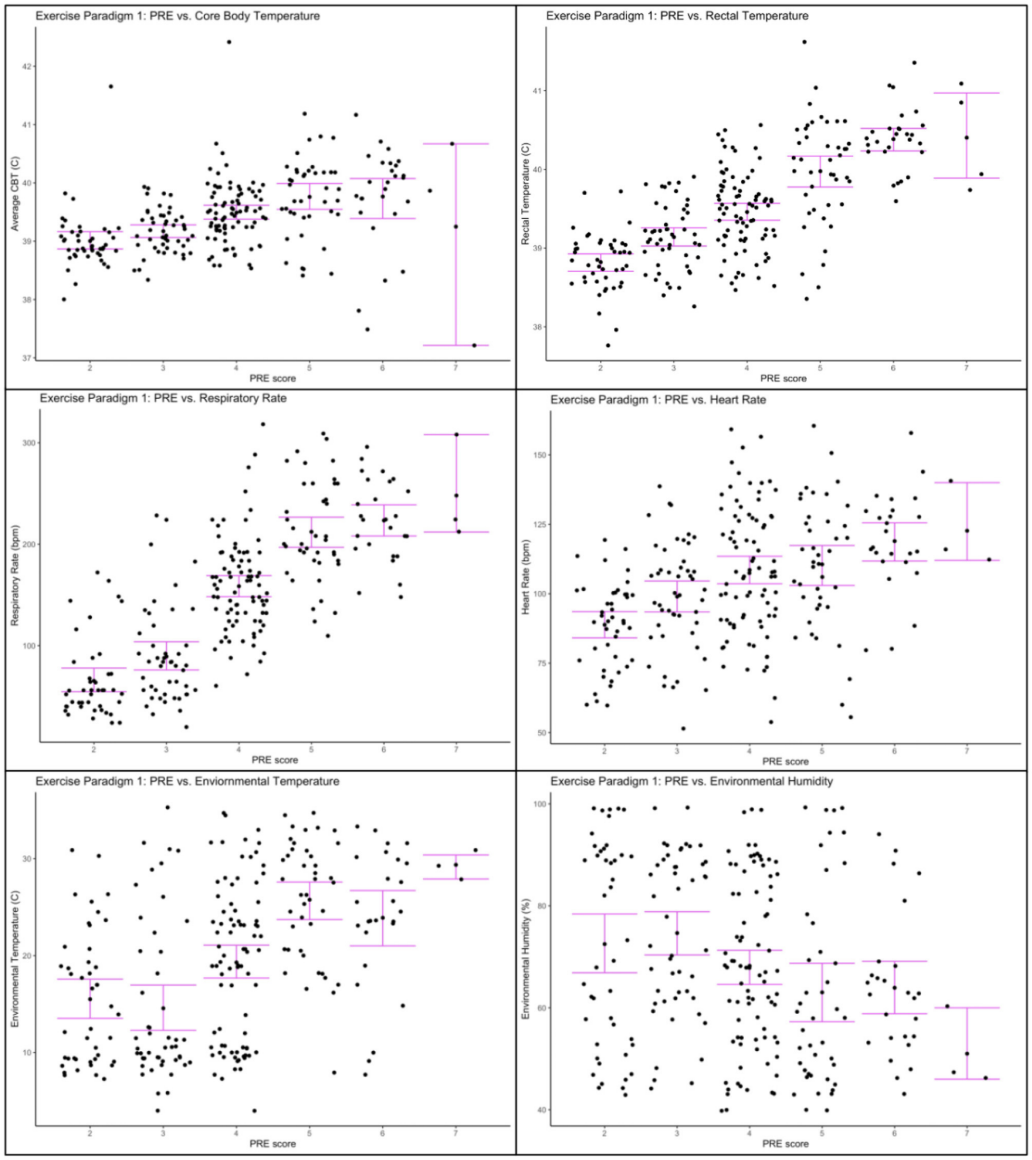
Physiological and environmental data collected during Exercise Paradigm 1 plotted against PRE score. **Upper left corner**: Core body temperature (°C) and PRE score; **Upper right corner**: Rectal temperature (°C) and PRE score; **Center left**: Respiratory rate (bpm) and PRE score; **Center right**: heart rate (bpm) and PRE score; **Lower left corner**: environmental temperature (°C) and PRE score; **Lower right corner**: environmental humidity (%) and PRE score. Error bars represent 95% confidence intervals.

Results of the regression models are given in Table 3. A mean one point change in PRE was associated with an increase in heart rate of 7.208 bpm ± 1.03 (*p* < 0.0001), increase in respiration rate of 44.66 bpm ± 2.37 (*p* < 0.001), increase in mean core body temperature of 0.17°C ± 0.04 (*p* < 0.001), increase in rectal temperature of 0.69°C ± 0.04 (*p* < 0.001), increase in environmental temperature of 3.01°C ± 0.38 (*p* = 0.001), and a decrease in environmental humidity −3.19% ± 0.86 (*p* = 0.0002). Figure 3 shows the relationship between each physiological and environmental parameter measured across PRE score.

**Table 3:** Regression model prediction of PRE by physiological and environmental measures for Exercise Paradigm 1.

**Variable**	**Regression coefficient ±standard error**	***P*-value**
Heart rate	7.21 ± 1.03 bpm	*p < * 0.0001
Respiratory rate	44.66 ± 2.37 bpm	*p < * 0.0001
Mean core body temperature	0.17 ± 0.04°C	*p < * 0.0001
Rectal temperature	0.69 ± 0.04°C	*p < * 0.001
Environmental temperature	3.01 ± 0.38°C	*p < * 0.001
Environmental humidity	−3.19 ± 0.86 %	*p =* 0.0002

### 3.2 Exercise paradigm 2

**Figure 4:**
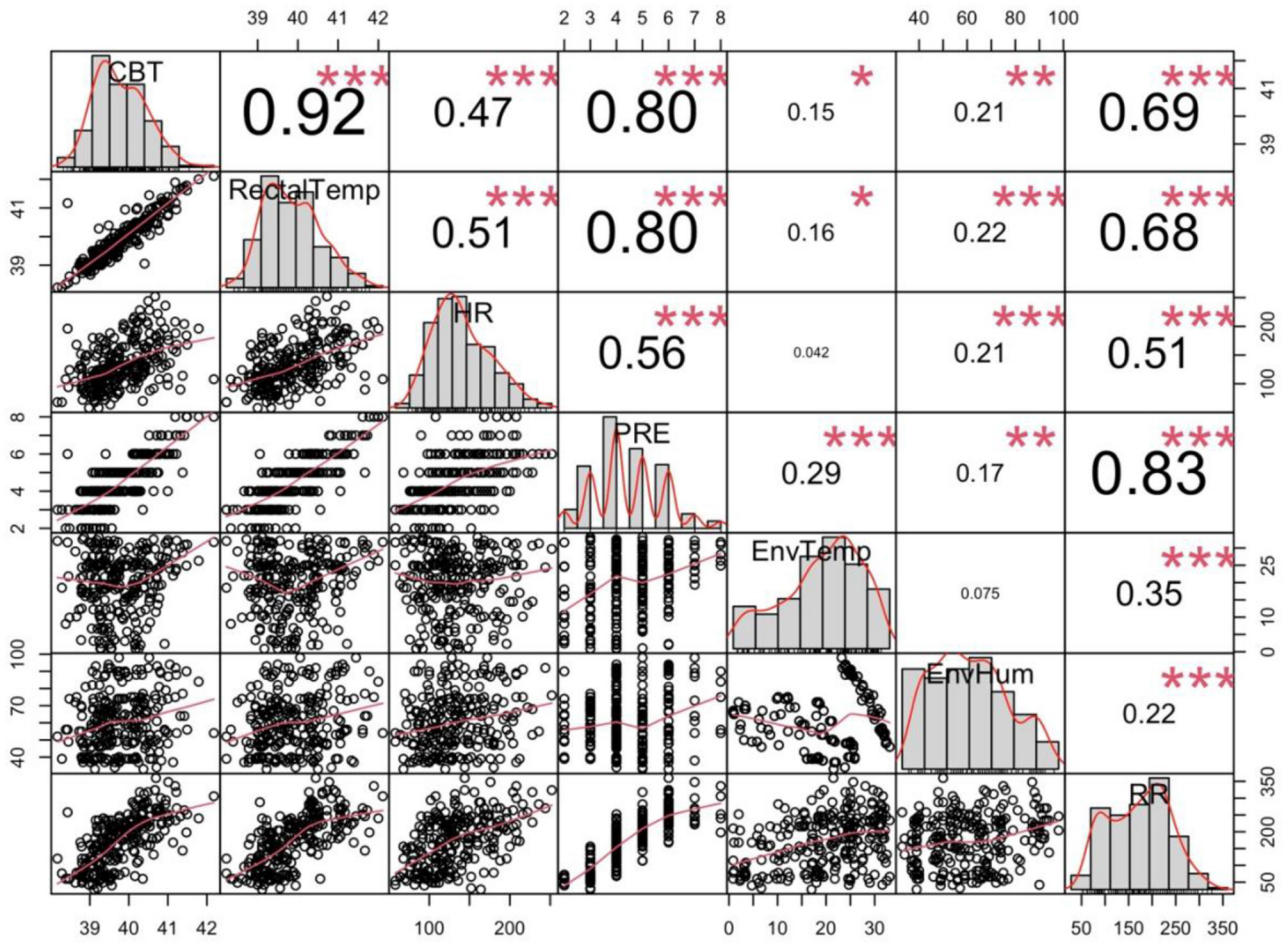
Correlation matrix of all the raw data for Exercise Paradigm 2. The average core body temperature (CBT—°C), rectal temperature (RectalTemp—°C), heart rate (HR—bpm), environmental temperature (EnvTemp—°C), environmental humidity (EnvHum—%), perceived respiratory (RR—bpm). *p ≤ 0.05, **p < 0.01, ***p < 0.001.

The interclass correlation for 20% of the double coded videos was 0.83 and for 20% of the triple coded videos was 0.83, indicating good reliability. A correlation matrix for data collected during Exercise Paradigm 2 is presented in Figure 4. There was a weak correlation between PRE score and environmental temperature (*r* = 0.29) and environmental humidity (*r* = 0.17). There was a moderate correlation between PRE score and heart rate (*r* = 0.56). There was a strong correlation between PRE score and core body temperature (*r* = 0.80), rectal temperature (*r* = 0.80) and respiratory rate (*r* = 0.83).

**Figure 5:**
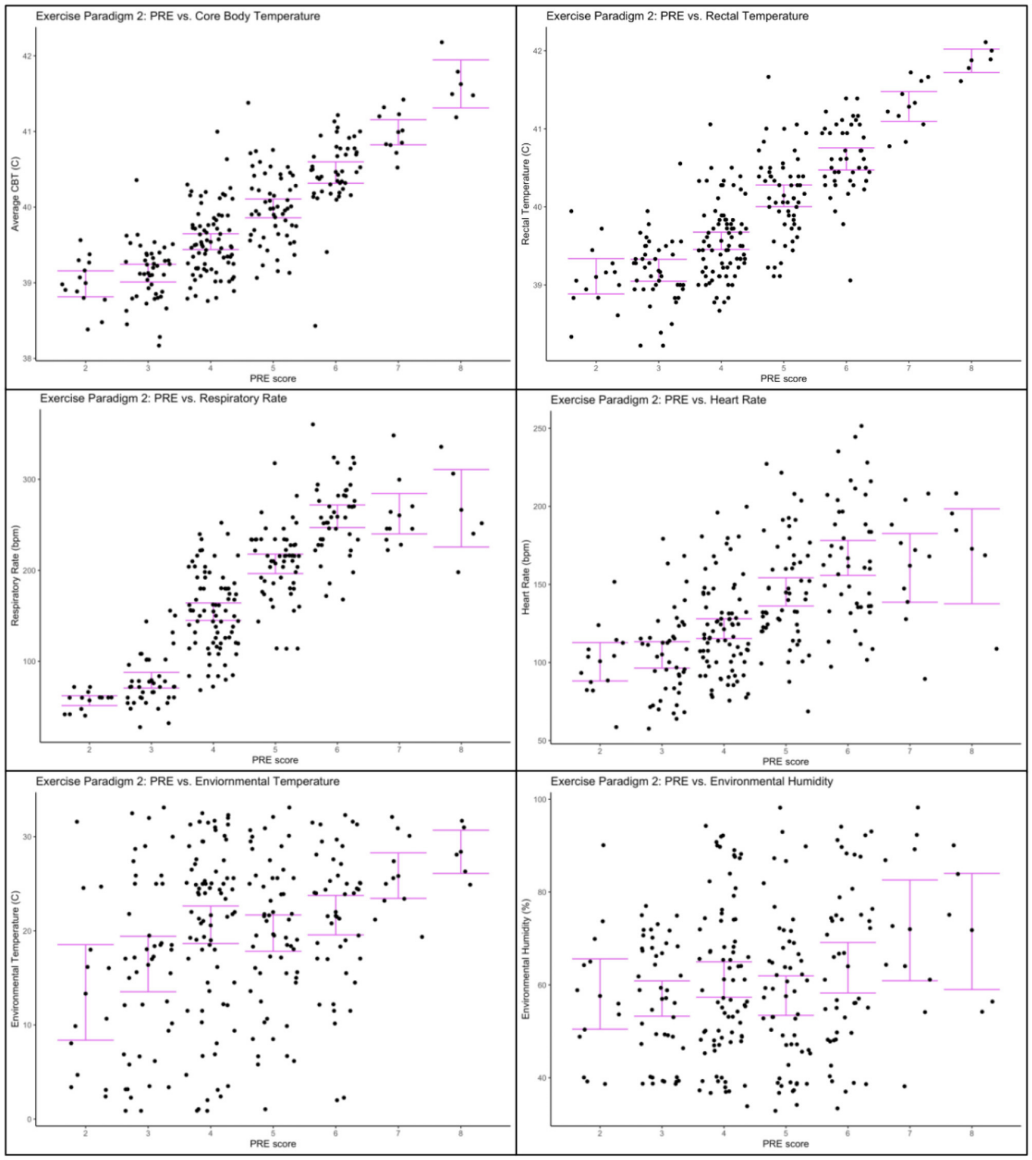
Physiological and environmental data collected during Exercise Paradigm 2 plotted against PRE score. **Upper left corner**: Core body temperature (°C) and PRE score; **Upper right corner**: Rectal temperature (°C) and PRE score; **Center left**: Respiratory rate (bpm) and PRE score; **Center right**: heart rate (bpm) and PRE score; **Lower left corner**: environmental temperature (°C) and PRE score; **Lower right corner**: environmental humidity (%) and PRE score. Error bars represent 95% confidence intervals.

Table 4 shows the regression coefficients for each dependent variable from the regression models. A mean one point change in PRE was associated with an increase in heart rate of 16.63 bpm ± 1.49 (*p* < 0.0001), increase in respiration rate of 46.5 bpm ± 2.06 (*p* < 0.001), increase in mean core body temperature of 0.45°C ± 0.02 (*p* < 0.001), increase in rectal temperature of 0.88°C ± 0.04 (*p* < 0.001), increase in environmental temperature of 1.89°C ± 0.39 (*p* < 0.001), and an increase in environmental humidity 2.08% ± 0.77 (*p* = 0.007) (see Table 4). Figure 5 shows individual graphs for Exercise Paradigm 2 for each physiological and environmental parameter measured across PRE score.

**Table 4:** Regression model prediction of PRE by physiological and environmental measures for Exercise Paradigm 2.

**Variable**	**Regression coefficient ±standard error**	***P*-value**
Heart rate	16.63 ± 1.49 bpm	*p < * 0.0001
Respiratory rate	46.50 ± 2.06 bpm	*p < * 0.0001
Mean core body temperature	0.455 ± 0.02°C	*p < * 0.0001
Rectal temperature	0.88 ± 0.04°C	*p < * 0.0001
Environmental Temperature	1.89 ± 0.39°C	*p < * 0.0001
Environmental Humidity	2.08 ± 0.77 %	*p =* 0.007

## 4 Discussion

The DeChant PRE scale was developed based on the CR-10 Borg scale (6, 7). Validity of the scale was confirmed by its positive correlations with several important physiological measures (i.e., core body temperature, rectal temperature, heart rate, and respiratory rate) in a working dog population in two exercise paradigms. Interestingly, the correlation coefficients for heart rate, core body temperature and rectal temperature were higher for Exercise Paradigm 2 than Exercise Paradigm 1. This may be due to the longer duration of Exercise Paradigm 2 which may have caused heart rate and temperature to be elevated longer, suggesting that the quantitative relationship between heart rate and temperature with PRE is moderated by the physical activity/exercise paradigm.

All results were consistent across both exercise paradigms except for the relationship between PRE and humidity. We observed a negative relationship in exercise paradigm 1 and a positive relationship in exercise paradigm 2. This may simply reflect the difference in daytime temperature and humidity relationships and seasonality. Figure 4 shows that higher humidity was associated with lower temperatures when we collected data for Exercise paradigm 1 (*r* = −0.26), explaining the negative relationship with humidity and PRE. Figure 5 show that in contrast, humidity was weakly associated with higher temperatures (*r* = 0.22) indicating the positive relationship between PRE and humidity.

The rectal temperature had a stronger correlation with PRE than core body temperature. This could be due to the timing of the data collection, because rectal temperature was measured at the exact time when PRE was scored, whereas core body temperature was averaged within a 5-min period. We averaged across a 5-min window to allow for slight differences in the timing between the core temperature system timestamp and the timestamp of the PRE video and rectal temperature. Future studies could better prioritize precise simultaneous recording of the measures.

Panting is one of the primary perceptually relevant and easy behaviors to monitor by handlers (24). One benefit of the DeChant PRE scale for dogs is a detailed description of panting and its changes with increases in exercise effort and subsequent PRE score increases. Because the DeChant PRE scale correlates with other physiological parameters such as core body temperature, rectal temperature, it may be useful to help predict when the dog may be approaching higher physiological measurements. However, this scale is not anticipated to be a replacement for taking the temperature of a dog.

The dogs that participated in this study were of one breed, physically fit, not pregnant, received regular exercise, and were deemed healthy by the owner. The DeChant PRE scale needs more research before it can be adapted or applied to broader populations of dogs of different breed and other demographics.

For detection dogs, olfactory performance may be diminished following strenuous exercise (25). When dogs are panting to lower their temperature via evaporative cooling, they are not able to close their mouth and thus, may not be able to sniff as thoroughly (24). As the DeChant PRE scale allows for quantitative scoring of dogs' respiratory effort, it may help handlers refine their evaluation of their dog's readiness for detection work. Nonetheless, detection performance was not part of this study and should be evaluated more thoroughly in future studies to determine if PRE is associated with reduced olfactory performance.

One limitation was the use of one mesocephalic breed, Labrador Retrievers, for data collection. Dolichocephalic and brachycephalic dog breeds demonstrate physiological as well as behavioral differences regarding thermoregulation. Specifically, brachycephalic breeds have greater propensity to develop heat related illness compared to mesocephalic and dolichocephalic breeds (26). Future research should explore other dog breeds as well as different exercise paradigms to collect more data on PRE scores.

Another limitation of this study was not having documented PRE scores on the extreme values of the scale (i.e., < 2 and >8. A score of “0” or “1” would be expected in dogs that were sleeping or resting. In addition, no scores of “9” or “10”, which would have indicated maximal effort, were recorded. However, as dogs with PRE scores of “9” or “10” may approach heat injuries, it was not within the scope of this research. It does, however, suggest that future research may further refine this scale into a more optimal 7- or 8-point scale rather than the 10-point scale proposed here.

When comparing our scale against the only other published scale for PRE in dogs (20), both scales showed good inter-observer agreement and correlation with physiological parameters. We observed a substantially higher correlation between heart rate, respiration rate and core temperature on our proposed scale. We anticipate this may be due to our scale providing more score options in the “moderate” to “maximal” range. In addition, the exercise paradigms used in our study were more strenuous and induced increased averages in our measured physiological parameters. For example, our mean HR post exercise was 118 bpm in Paradigm 1 and 153 bpm in Paradigm 2 vs. 90 bpm in Swanson et al. (20).

Overall, the results suggest that the DeChant PRE scale may be a simple visual scale to measure dog respiratory effort. Our analysis with over 70 dogs indicates that it can be scored reliably, and is significantly associated with environmental temperature, core body temperature, rectal temperature, heart rate and respiratory rate. Future work is needed to extend these results to more breeds and to conduct validation for use as a measure to predict detection performance or heat injury risk.

## Data Availability

The raw data supporting the conclusions of this article will be made available by the authors, without undue reservation.

## Footnotes

^1^Diet A: Guaranteed analysis: Crude Protein (min.) 21.0%, Crude Fat (min.) 13.0%, Crude Fiber (max.) 3.6%, Moisture (max.) 10.0%, Eicosapentaenoic Acid (EPA) (min.) 0.17%, Docosahexaenoic Acid (DHA) (min.) 0.07%, Vitamin E (min.) 420 IU/kg, Ascorbic acid^*^ (min.) 240 mg/kg, Glucosamine^*^ (min.) 500 mg/kg, Chondroitin sulfate^*^ (min.) 4 mg/kg. Ingredient list: brewers rice, chicken by-product meal, corn, animal fat, natural flavors, wheat gluten, fish oil, dried plain beet pulp, pea fiber, vegetable oil, calcium carbonate, potassium chloride, powdered psyllium seed husk, sodium silico aluminate, sodium hexametaphosphate, DL-methionine, fructooligosaccharides, trace elements, vitamins. Diet B: Guaranteed analysis: Crude Protein (min.) 30.0%, Crude Fat (min.) 20.0%, Crude Fiber (max.) 4.1%, Moisture (max.) 10.0%, Leucine (min.) 1.74%, Methionine (min.) 0.5%, Eicosapentaenoic Acid (EPA) + Docosahexaenoic Acid (DHA) = 0.25% (min.), Vitamin E (min.) 490 IU/kg, Ascorbic acid^*^ (min.) 240 mg/kg, Lutein^*^ (min.) 14 mg/kg, Beta-carotene^*^ (min.) 2 mg/kg, Glucosamine^*^ (min.) 500 mg/kg, Chondroitin sulfate^*^ (min.) 4 mg/kg. Ingredient list: chicken by-product meal, corn, brewers rice, animal fat, wheat gluten, corn gluten meal, natural flavors, dried plain beet pulp, pea fiber, fish oil, vegetable oil, sodium silico aluminate, calcium carbonate, potassium chloride, powdered psyllium seed husk, trace elements, vitamins.

## Supplementary material

Supplementary Video 1 Demonstration of PRE score 2 in Labrador Retriever: Light.

Supplementary Video 2 Demonstration of PRE score 3 in Labrador Retriever: Moderate.

Supplementary Video 3 Demonstration of PRE score 4 in Labrador Retriever: Somewhat heavy.

Supplementary Video 4 Demonstration of PRE score 6 in Labrador Retriever: Moderately heavy.

Supplementary Video 5 Demonstration of PRE score 8 in Labrador Retriever: Very very heavy.

Supplementary Video 6 Demonstration of PRE score 5 in Labrador Retriever: Heavy.

Supplementary Video 7 Demonstration of PRE score 7 in Labrador Retriever: Very heavy.
